# Highly Altered State of Proton Transport in Acid Pools in Charged Reverse Micelles

**DOI:** 10.1021/jacs.2c11331

**Published:** 2023-01-12

**Authors:** Hongxia Hao, Ellen M. Adams, Sarah Funke, Gerhard Schwaab, Martina Havenith, Teresa Head-Gordon

**Affiliations:** † Kenneth S. Pitzer Center for Theoretical Chemistry, Department of Chemistry, University of California, Berkeley, California94720, United States; ‡ Cluster of Excellence Physics of Life, Technische Universität Dresden, 01307Dresden, Germany; § Helmholtz-Zentrum Dresden-Rossendorf, Institute of Resource Ecology, 01328Dresden, Germany; ∥ Lehrstuhl für Physkalische Chemie II, Ruhr Universität Bochum, 44801Bochum, Germany; ⊥ Department of Bioengineering, Department of Chemical and Biomolecular Engineering, University of California, Berkeley, California94720, United States; # Chemical Sciences Division, Lawrence Berkeley National Laboratory, Berkeley, California94720, United States

## Abstract

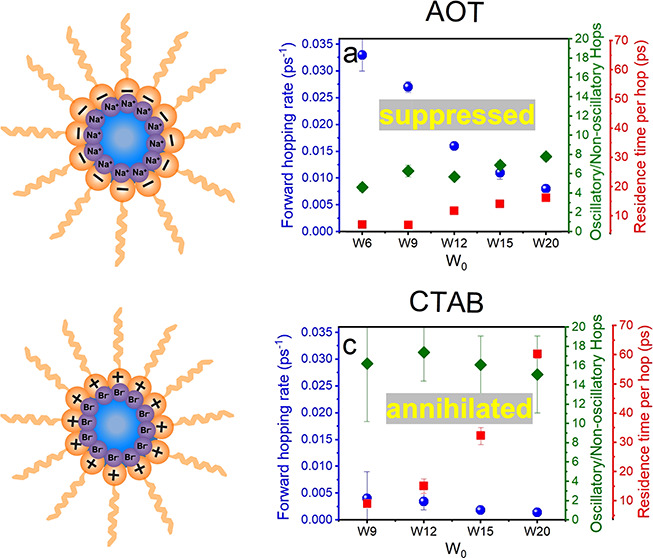

Transport mechanisms of solvated protons of 1 M HCl acid pools, confined within reverse micelles (RMs) containing the negatively charged surfactant sodium bis(2-ethylhexyl) sulfosuccinate (NaAOT) or the positively charged cetyltrimethylammonium bromide (CTABr), are analyzed with reactive force field simulations to interpret dynamical signatures from TeraHertz absorption and dielectric relaxation spectroscopy. We find that the forward proton hopping events for NaAOT are further suppressed compared to a nonionic RM, while the Grotthuss mechanism ceases altogether for CTABr. We attribute the sluggish proton dynamics for both charged RMs as due to headgroup and counterion charges that expel hydronium and chloride ions from the interface and into the bulk interior, thereby increasing the pH of the acid pools relative to the nonionic RM. For charged NaAOT and CTABr RMs, the localization of hydronium near a counterion or conjugate base reduces the Eigen and Zundel configurations that enable forward hopping. Thus, localized oscillatory hopping dominates, an effect that is most extreme for CTABr in which the proton residence time increases dramatically such that even oscillatory hopping is slow.

## Introduction

The movement of protons within an aqueous environment is essential to a multitude of (bio)chemical and physical processes, including catalysis,^1^ ATP synthesis,^2^ transmembrane potentials,^3^ aerosol properties,^4−6^ and energy technologies such as fuel cells.^7,8^ Although the unusual dynamics of the solvated proton had been introduced over two centuries ago,^9^ many new facets of its fundamental properties remain a topic of fascination and current debate.^7,10−26^ For instance, protons are known to propagate through pure water much faster than other ions via the Grotthuss mechanism,^7,9,10,14,21,27−30^ in which a proton hops along in the forward direction from one water to the next via the specific presence of hydrogen-bonding configurations involving Eigen and Zundel sequences (the special partner dance).^27,28,30−35^ While the experimental and theoretical studies of proton transport mechanism in bulk acid water solutions continue to mature in more detail,^18,19,32−36^ most real-world systems in which proton transport plays an essential role actually occurs within confined and/or complex interfacial water environments. Within this regime, protons are thought to diffuse along the surface boundary of the air–water,^37−39^ solid–water,^25^ or membrane boundaries^40^ via water-mediated interactions. Considering that many interfaces of chemical and biophysical interest contain many polar and charged groups, this brings into question how the chemical nature of the confining interface influences proton solvation dynamics.^26^

Confinement of water is known to alter its hydrogen-bonding structure and dynamics, resulting in a weaker hydrogen-bonding network with slower dynamics.^41,42^ Reverse micelles (RMs) make an excellent model system to study confinement and interfacial effects as inner surface properties such as charge can be easily manipulated by changing the amphiphilic lipid headgroup.^5,22,41−50^ In addition, the size of the nanoscopic water pool can be altered through the relation *W*_0_ = [H_2_O]/[lipid], allowing properties of water in interfacial vs bulk-like limits to be explored,^41,50^ as well as proton concentration changes due to pH.^51^ The effect of confinement on proton transport within charged RMs was first investigated through the photoinduced acid dissociation of the fluorescent photoacid 8-hydroxypyrene-1,3,6-trisulfonate (HPTS).^52−57^ HPTS molecules in anionic and nonionic RMs demonstrated reduced photoexcited deprotonation due to a confinement effect, while no deprotonation occurs in cationic RMs owing to partitioning of the HPTS photoacid into the micellar interface. Recent studies from the Bakker group have investigated the solvation structure and proton transport of confined acid in negatively charged surfactant bis(2-ethylhexyl) sulfosuccinate with a sodium counterion (NaAOT), or the positively charged cetyltrimethylammonium surfactant with a bromide countercharge (CTABr) RMs.^58,59^ Infrared transient absorption spectroscopy revealed a four times slowdown in the anisotropy of 7 M HBr confined in CTABr RMs (*W*_0_ = 12–40, *r* = 2.2–7.4 nm) relative to the bulk liquid (∼1.6 ps), and the anisotropy became increasingly slower with decreasing micelle size. Further pump–probe measurements determined that protons confined in CTABr mostly have an asymmetric hydration structure, in which one of the three hydrogen bonds of H_3_O^+^ forms a weaker hydrogen bond with respect to the other two.^59^ These results were compared to the hydration structure formed in AOT RMs, where it was concluded that hydrated protons complex to the RSO_3_^–^ headgroup of the AOT surfactant. Similar values were observed for less concentrated 3 M HBr RMs, leading to the conclusion that the high acid concentration inside of the micelles was not the source of the slowdown but rather came from an interfacial or confinement effect.^58^ It should be noted, however, that the AOT micelles were only probed in the small size range (*W*_0_ = 1–3); in this regime, it can be estimated that approximately 66–90% of water in the RMs is interfacial.

Recently, we have investigated proton transport mechanisms in concentrated (1 M) HCl acid pools contained within larger RMs assembled from nonionic IGEPAL surfactants.^60^ Using TeraHertz (THz) and dielectric relaxation (DR) spectroscopy and analyzing them with the ReaxFF-CGeM reactive force field simulations enabled, for the first time, to characterize concentrated acid pools that match experimental conditions. We identified a change in mechanism from Grotthuss forward shuttling to one that favors local oscillatory hopping within the nonionic RM.^61^ This is due to a preference for high concentrations of H_3_O^+^ and Cl^–^ ions to adsorb to the reverse micelle interface, causing a “traffic jam”, in which the short-circuiting of the hydrogen-bonding motif of the hydrated hydronium ion decreases the forward hopping rate. Similar conclusions were reached in a related computational study on the same nonionic RMs that demonstrated that an excess proton seeks out the water interface.^22^ However, while the forward proton hopping rate for a single excess proton increases as system size increases,^22^ we found that at higher 1 M acid concentrations, the forward proton hopping rate per hydronium decreases, while the local oscillatory hopping (hopping pattern 1 → 2 → 1) increases with increasing the RM size.^60^

**Figure 1:**
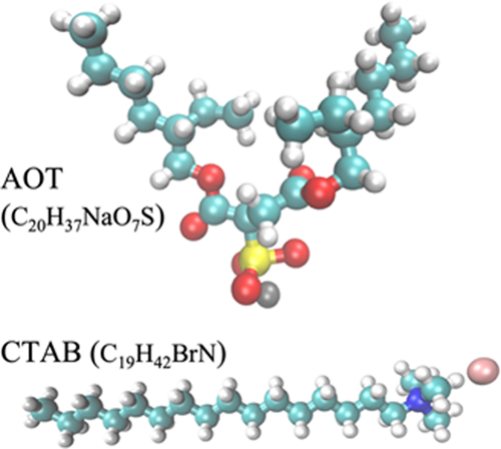
Structure of the charged surfactant molecules with different headgroup charges. NaAOT has a negatively charged sulfonic acid headgroup with a sodium counterion, and CTABr contains a positively charged trimethylammonium headgroup with a bromide counterion.

Here, we consider whether and how the mechanism of proton hopping in acidic water pools within RMs changes from IGEPAL as we vary the charge of the lipid headgroup and its counterion that defines different chemistry at the interface beyond simple confinement.^44^ An experimental comparison of how the interface charge influences proton transfer in RMs of larger RM sizes does not yet exist, due to limitations in the distribution of fluorescent probes and the ability to produce RMs with highly concentrated acids. We have demonstrated below that we are able to produce label-free anionic and cationic RMs with concentrated 1 M HCl with both NaAOT and CTABr (Figure 1). The properties of the solvated protons in these systems were probed as a function of the acid pool size with THz absorption and DR spectroscopies and simulated with the reactive force field ReaxFF-CGeM,^62,63^ which we show reproduces the experimental observations well. Further experimental and theoretical and simulation details are provided in the Materials and Methods section and Supporting Information.

We find that the surfactant charged headgroup and its counterions create a congested ionic interface that slows down the proton dynamics dramatically compared to the nonionic RM. The lipid headgroup and counterions of NaAOT help stabilize the solvated proton near the micelle interface even more than that for IGEPAL, resulting in an even greater reduction in forward hopping events to instead favor localized oscillatory hopping. More dramatically, we find that the Grotthuss mechanism is completely suppressed for the CTABr system, with long proton residence times that slow down even the oscillatory hopping mechanism, no matter how large the reverse micelle is. Counterintuitively, this is not due to strengthening/weakening of the hydrogen-bonding network by the particular surfactant headgroup, where it would be expected that complexation and/or repulsion of the hydrated proton to the headgroup would be a major driving force. Rather, our results reveal that the counterions associated with these surfactants are responsible for proton transport suppression. These ions disrupt the hydrogen-bonding network and compete with protons for solvation water, ultimately limiting the pathways that protons can take and increasing their residence time at a particular water molecule. Whereas (inhibited) proton hopping typically occurs along the interfacial boundary via water-mediated interactions for the nonionic RM, the charged systems lose available water to general ion solvation that disrupts the formation of the Eigen and Zundel sequences^7,16,18,20,24,28,30,36,64^ that are needed for Grotthuss shuttling.

## Results

To support and quantify these molecular descriptions, we first consider THz and DR spectroscopy measurements taken on the charged RMs as a function of size. In the THz regime, spectral features corresponding to solvated ions can be isolated, where for the reverse micelles, this is achieved by subtracting the absorption spectrum of pure water RMs from the absorption spectrum of HCl containing RMs of the same size, as shown in eq 1.
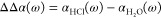
1It should be noted that in the case of NaAOT, an additional subtraction of *W*_0_ = 0 was performed since NaAOT spontaneously forms RMs in the absence of water, while CTABr does not. For simplicity, we will refer to all such difference spectra as ΔΔα.

**Figure 2:**
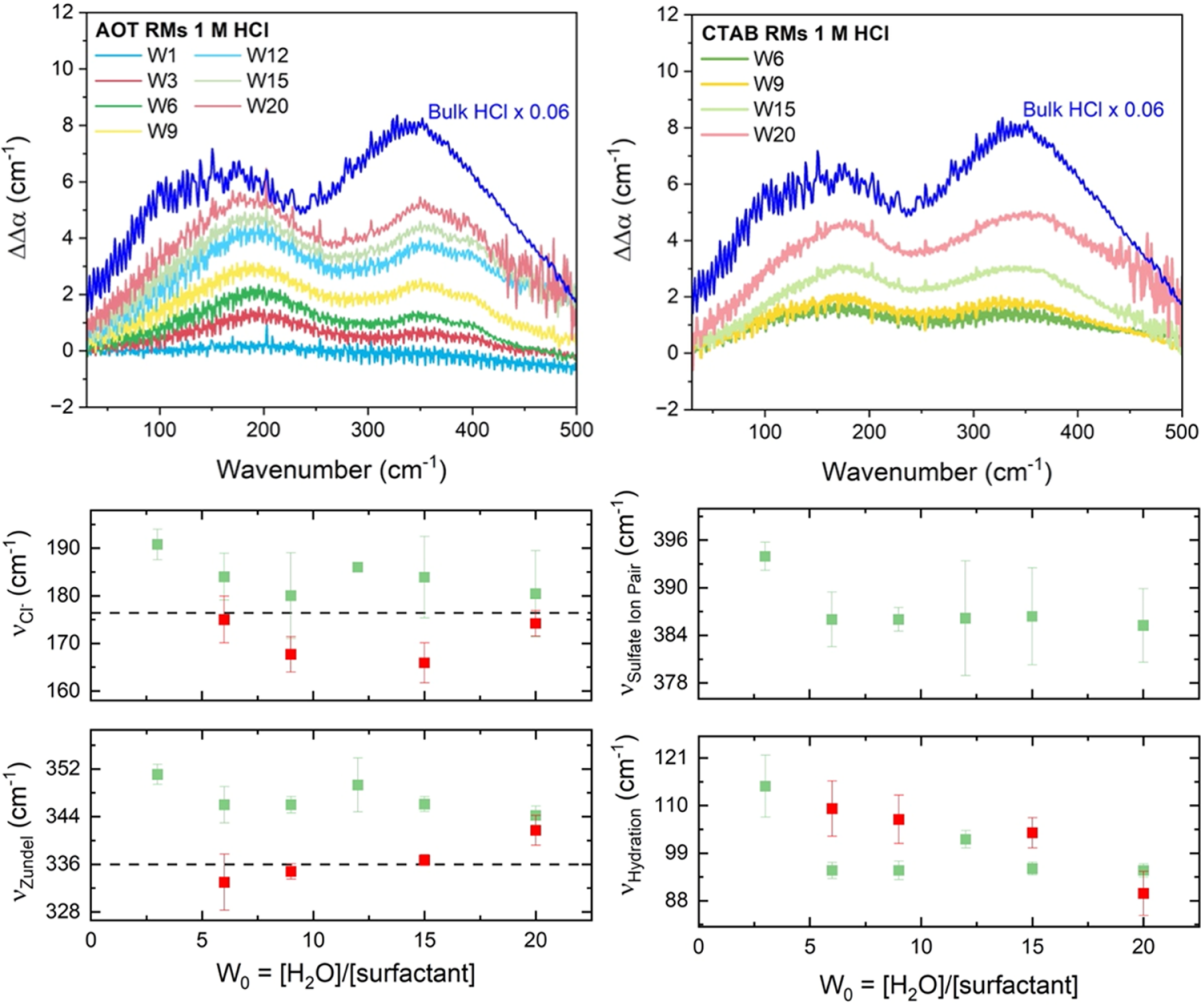
THz-FTIR spectra and spectral decompositions of negatively charged NaAOT and positively charged CTABr reverse micelles filled with 1 M HCl. (Top) Double difference absorption in the THz as a function of acid pool size in the NaAOT and CTABr reverse micelles. A spectrum of HCl in bulk solution is shown as the blue line for reference. (Bottom) Central peak frequencies of specific solvated ion modes for NaAOT (green) and CTABr (red) from spectral fitting with damped harmonic oscillators. Dashed lines represent the central peak frequencies of HCl in bulk solution. Further information on spectral decompositions is provided in the Supporting Information.

Figure 2 shows the ΔΔα corresponding to dissociated H_3_O^+^ and Cl^–^ ions confined in RMs composed of either NaAOT or CTABr, along with the center peak frequencies determined from spectral decomposition analysis for each RM size to better understand their underlying compositions (see the Supporting Information and Figure S1). For NaAOT, two spectral features are observed near ∼180 and ∼350 cm^–1^, which increase in intensity with increasing micelle size, while for CTABr, these same spectral peaks only grow at the larger RM sizes of W15 and W20. Previous studies have determined the 350 cm^–1^ peak and the low-frequency harmonic oscillator at ∼110 cm^–1^ in the THz decomposition correspond to hydration water of the solvated proton.^16^ The 350 cm^–1^ peak was previously assigned to the solvated Eigen complex, but a recent study found that a coupled motion of excess protons to the oxygen–oxygen vibration of the Zundel cation is responsible for that feature.^65^ Additionally, this same study revealed that ∼110 cm^–1^ component stems from the average waiting time between two consecutive proton transfer events, denoted as the transfer waiting time, τ_TW_. The 180 cm^–1^ peak has been previously ascribed to the rattling of Cl^–^ ions in their water cages,^16,66,67^ indicating that the Cl^–^ ion has a more extended aqueous solvation environment in the NaAOT reverse micelle, although that spectral signature is not evident until W15 before becoming especially prominent at W20 for the CTABr reverse micelles. This is different than the case of the RMs composed of the nonionic surfactant IGEPAL in which this peak is suppressed, and it was therefore concluded that Cl^–^ ions adsorb to the reverse micelle interface in that study.^61^ The weak 400 cm^–1^ peak for the NaAOT RM stems from the positively charged proton with the sulfonic headgroup of the surfactant,^68^ in line with a recent transient absorption study that found indirect evidence of proton–sulfate solvated ion pairs in NaAOT micelles.^59^ It should be noted that peaks corresponding to the surfactant counterions, Na^+^ and Br^–^,^66,69^ for NaAOT and CTABr, respectively, were not observed, indicating that the distribution of counterions in the RMs is the same with or without the presence of HCl. The spectral decompositions for NaAOT and CTABr show that the mobile acid ions, especially the 350 cm^–1^ and the 110 cm^–1^ peak, have perturbed THz signatures, indicating that the modes for H_3_O^+^ and Cl^–^ ions are surface sensitive and therefore show differences with respect to headgroup charge and counterions.

**Figure 3:**
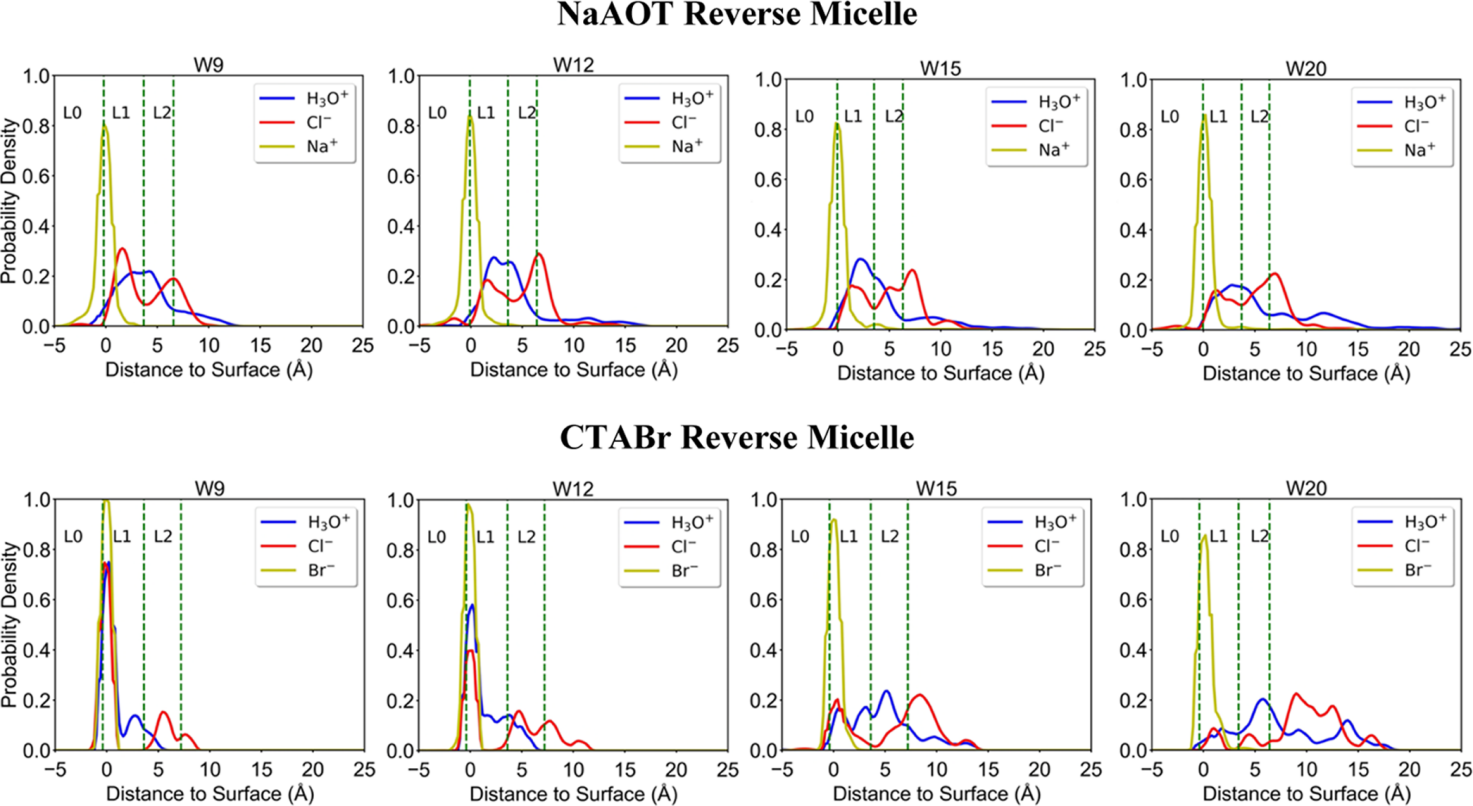
Probability density distribution of counterions, hydrated protons, and chloride ions in 1 M HCl reverse micelles of varying sizes. Interfacial layers are defined using an instantaneous surface method.^72^ Layers up to L2 do not have bulk-like numbers of hydrogen bonds and thus can be considered as interfacial areas. (Top) Ion densities from surface to interior for NaAOT. (Bottom) Ion densities from surface to interior for CTABr. Conclusions drawn from the ion probability densities are consistent with the radial distribution functions provided in Supporting Information Figure S2.

Turning to simulation, Figure 3 shows the ion probability densities of the NaAOT system with the aqueous 1 M HCl acid pools as simulated with the ReaxFF-CGeM model. Most of the protons and Cl^–^ ions adsorb to the interfacial region for NaAOT, but they are made more solvent-exposed by first layering against the counterion Na^+^ layer that remains strongly associated with the sulfonic headgroup, consistent with the 400 cm^–1^ assignment of the proton–sulfate solvated complex. The Cl^–^ ions have a bimodal distribution with one layer near the counterion and protons and another bleeding into the interior of the RM, also in agreement with the spectral signatures displayed in Figure 2. In the case of CTABr, when the system size is smaller than W12, all Br^–^ ions, Cl^–^ ions, and protons accumulate strongly at the interface with minimal solvent exposure. With an increase of system size to W15, however, protons start to spread to the outer surface layer and into the inner water pool with the conjugate base, again consistent with the THz amplitude trends evident in Figure 2. A similar effect was observed near lipid bilayers by Yamashita and Voth in which proton trapping by the headgroups and proton “escape” into the more bulk-like region was observed^70^ and later supported by multiple experiments.^71^ Overall, the charged interfaces increase the effective pH in the RM interior relative to that observed for nonionic surfactants.

**Figure 4:**
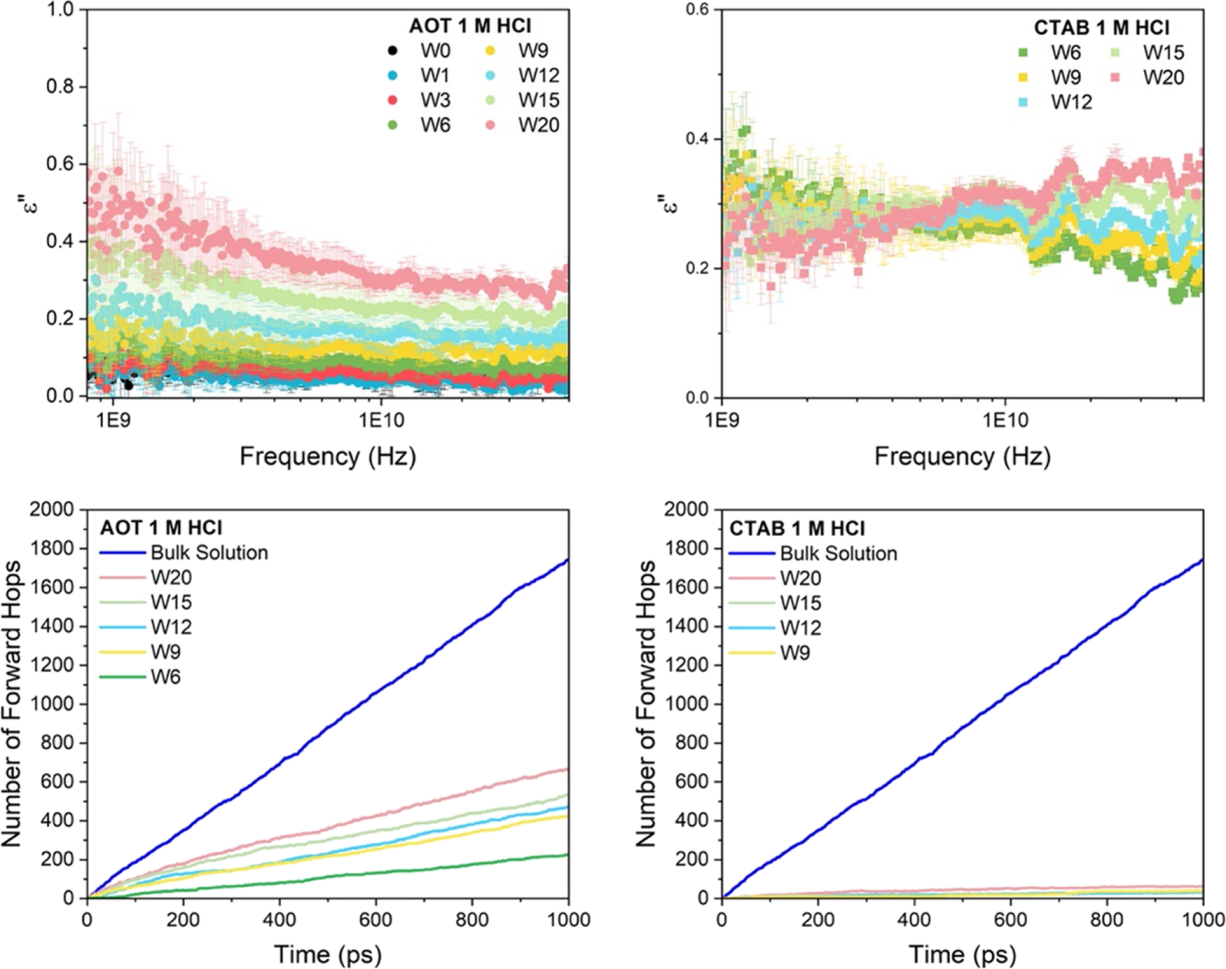
Evidence for diminishment of the Grotthuss hopping mechanism in reverse micelles of different charge. (Top) Imaginary permittivity, ε″, of (left to right) NaAOT and CTABr RMs of various sizes filled with 1 M HCl. (Bottom) Number of forward proton hops (without oscillatory motion) calculated from an MD trajectory from each RM system and a bulk system with 1 M HCl solution. See the SI for Debye fitting details and full results.

Since the THz absorption spectra and simulations show that the distribution of ions and solvation properties are influenced by the RM charge, it suggests that the dynamics of solvated protons may be impacted as well. The DR complex permittivity, ε(ω) = ε′ – iε″, is comprised of an imaginary part, ε″ (Figure 4) and a real component ε′ (Figure S3). It can be modeled as a sum of Debye modes with an additional term to account for the conductivity of the solution, as shown in eq 2.
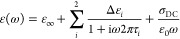
2where ε_∞_ is the permittivity at the high-frequency limit, Δε_*i*_ is the permittivity amplitude, τ_*i*_ is the rotational relaxation timescale of the *i*th process, σ_DC_ is the conductivity, and ε_0_ is the vacuum permittivity. Here, two Debye modes were found to best represent the DR data. The fast and slow rotational reorientation relaxation times of water determined from the ReaxFF-CGeM model are in very good agreement with the corresponding fitted quantities extracted from the experimental NaAOT and CTABr complex permittivities as a function of RM size (Figure S4).

However, the most informative experimental feature for the proton hopping mechanism is determined by the low-frequency region of ε″ of the complex permittivity spectra, which is dominated by a conduction band, which we have previously shown is directly interpretable as evidence of the number of forward hopping events without consideration of any unproductive oscillatory hopping (Figure 4).^61^ Hence the number of forward hops in the system without consideration of oscillatory hopping decreases with decreasing RM size, consistent with suppression of proton diffusion with increasing confinement.^22,58^ While the NaAOT RMs display similar behavior to IGEPAL, i.e., an increase in the low-frequency side of the spectrum with increasing micelle size is observed, the conductivity and thus the number of Grotthuss forward hopping events are greatly suppressed, given the nearly 10X difference in scale (see Figure 3b in ref (61)). Conversely, it can be seen for the CTABr RM that the HCl has no impact on the imaginary permittivity spectrum, and there is no measurable solution conductivity, which is also reproduced by simulations in the complete lack of forward hopping events.

**Figure 5:**
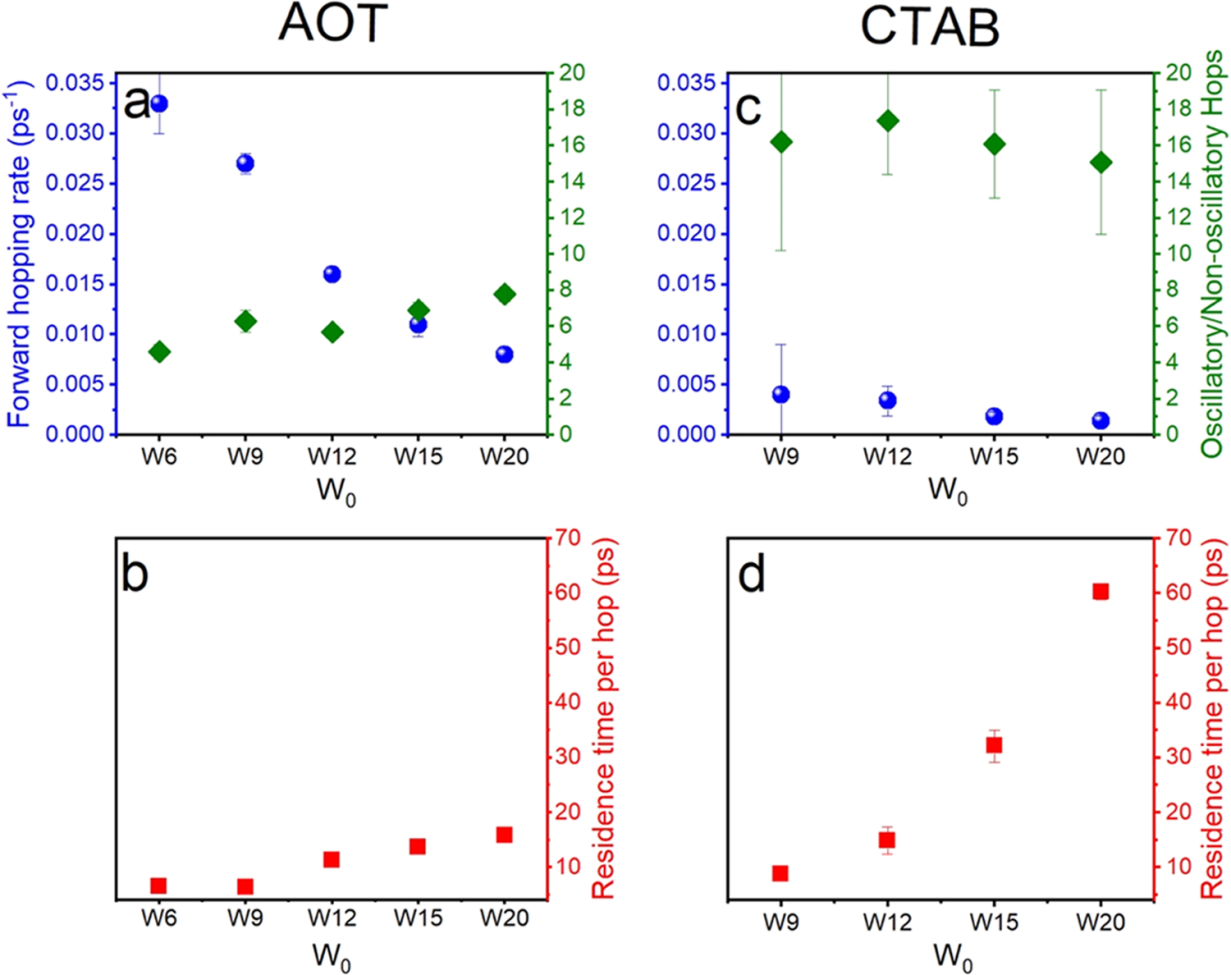
Mechanistic analysis of proton dynamics NaAOT and CTABr as a function of reverse micelle size from simulation. Forward proton hopping rates per hydronium (blue dot), ratio of oscillatory to nonoscillatory hops (green square), for (a) NaAOT and (c) CTABr. Residence time per hop (red square) for (b) NaAOT and (d) CTABr.

Different from the number of forward hopping events with no oscillatory hopping, we next measure the forward hopping rate or frequency, which is the number of forward hopping events per hydronium. Like IGEPAL, which undergoes a transition at W9 to a rate controlled by oscillatory hopping, the NaAOT reverse micelle also transitions from forward hopping to an oscillatory hopping mechanism at W15 (Figure 5a), which is accompanied by an increase in the residence time per hop of the proton (Figure 5b). This is consistent with the red shift of the ∼110 cm^–1^ peak, which is attributed to an increase in proton residential time scales.^65^

In contrast, CTABr RMs do not display a change in proton transport mechanism at any reverse micelle size; protons preferentially move via a local oscillatory hopping mechanism and have significantly larger residence times compared to NaAOT, again consistent with the red shift of the 110 cm^–1^ peak observed in recent work. For CTABr, the protons are freed from the crowdedness of the interface as the system size increases, but the protons remain associated with the chloride ions in the bulk interior, such that even the local oscillations are slower.

## Discussion and Conclusions

We have previously found that the suppression of Grotthuss shuttling for nonionic reverse micelles is due to a jamming effect of the high proton concentrations at the interface, such that forward proton transfer events are greatly diminished, leading to increases in oscillatory hopping instead.^61^ However, different classes of reverse micelle surfactants and counterions will give rise to different molecular interactions (Coulombic field effects, hydrogen bond interactions, ion pairing, etc.) that will in turn affect spatial distribution functions, dynamical signatures such as proton transfer waiting times, as well as mechanistic outcomes such as the relative role of proton rattling vs Grotthuss proton shuttle.

We have shown that proton transport in a confined environment is strongly influenced by the charged interfaces, with a complex interplay of the headgroups and their counterions determining the diminishment of Grotthuss forward hopping. Suppression of proton transport has been largely attributed to localization of the proton at the interfacial region of the confined environment regardless of headgroup.^22,37,38,61^ This is not the case for the charged RMs with either cationic or anionic headgroups, as shown here, where the corresponding counterions are essential to explain why this is not strictly an interfacial effect. Furthermore, the possibility that proton diffusion could be enhanced through interaction with the anionic SO_3_^–^ groups to form SO_3_H to serve as an intermediary for proton hopping, as observed in polyelectrolyte membrane PEM mimics,^73−77^ is also not observed here. Rather, the requirement of a counterion to maintain the integrity of a reverse micelle organization reduces the surface sites, thereby forcing the H_3_O^+^ and the Cl^–^ conjugate base to move to the outer reaches of the interface (NaAOT) or to diffuse into the bulk-like interior (CTABr).

In both cases, the acid pool of charged RMs has lower pH compared to the nonionic RMs, with available water devoted to solvating more and more ions at the expense of forming the extended solvated hydronium complexes needed for a productive proton transfer. Hence, the Grotthuss forward hopping is greatly diminished for NaAOT and annihilated for CTABr because the proton traffic jam is no longer localized at the surfactant interface as in the case of the nonionic RM. Instead, the proton traffic jam is now ubiquitously found throughout the interior of the charged reverse micelles and their more acidic water pools in which protons oscillate locally and with longer waiting times relative to the nonionic RMs which have fewer ions and more neutral encapsulated water pools.

## Materials and Methods

### Reverse Micelle Preparation

Sodium bis(2-ethylhexyl) sulfosuccinate (NaAOT), cetyltrimethylammonium bromide (CTABr), cyclohexane, 1-hexanol, and hexane were used without further purification. A 1 M HCl solution was prepared by dilution of concentrated HCl (37% v/v). Negatively charged RMs were created by dissolving NaAOT in cyclohexane for a 0.1 M stock solution. An appropriate amount of water or 1 M HCl solution was then added to make sizes varying from *W*_0_ = 1 to 20. Positively charged RMs were formed with a 3:1 CTABr/1-hexanol mixture and water (or 1 M HCl) in hexane solvent. CTABr sizes of *W*_0_ = 6–20 was prepared. All RM samples were sonicated to ensure complete emulsification.

### THz-FTIR Spectroscopy

A commercial FTIR spectrometer (Bruker, Vertex 80v) equipped with an external Hg lamp and a Si-bolometer detector was utilized to collect THz spectra (30–680 cm^–1^). Samples were loaded in a demountable diamond cell with an effective sample thickness of 0.5 mm. The sample solution was held at a constant temperature of 20 °C (± 0.5 °C) with an external chiller. The sample chamber was continuously purged to remove water vapor. Spectra are an average of 64 scans with 1 cm^–1^ resolution and were reproduced at least three times. Average error of the measurements is 5 cm^–1^. The frequency-dependent absorption coefficient, α, of the sample was determined from the Beer–Lambert law, as shown in eq 3.
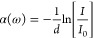
3where *d* is the sample thickness and *I* and *I*_0_ are the intensities transmitted through the sample and reference.

### Dielectric Relaxation Spectroscopy

The complex permittivity of the samples was measured over the range 1–50 GHz with a microwave network analyzer (Agilent Technologies, N5235A PNA-L, Santa Clara) with a slim form probe. The probe was calibrated to air/short/*W*_0_ = 0 of IGEPAL CO-520. Samples were equilibrated to a temperature of 20 °C with a thermal bath prior to measurement.

### Computational Details

Reverse micelles with varying sizes were prepared according to the water-to-surfactant molar ratio, *W*_0_ = [H_2_O]/[surfactant], an estimate of the size of the water pool with the corresponding number of surfactants in the reverse micelles. Here in our simulation, the number of surfactants (NaAOT and 3:1 CTABr/1-hexanol, respectively) was estimated according to *W*_0_ after the number of waters was determined based on the experimentally measured water pool radius. The corresponding radii of the water pool in NaAOT micelles are 1.42 nm (*W*_0_ = 6), 1.86 nm (*W*_0_ = 9), 2.29 nm (*W*_0_ = 12), 2.73 nm (*W*_0_ = 15), and 3.19 nm (*W*_0_ = 20).^43^ For CTABr micelles, the water pool radii are 1.17 nm (*W*_0_ = 9), 1.56 nm (*W*_0_ = 12), 1.95 nm (*W*_0_ = 15), and 2.60 nm (*W*_0_ = 20). The remainder of the simulation box was filled with solvent molecules (cyclohexane in NaAOT micelle and hexane in CTABr micelle) at a bulk density of 298 K. The number of solvent molecules in each system was chosen to have at least a total mass in solvent greater than 80%. Detailed compositions of each *W*_0_ system for the reverse micelle systems can be found in Supporting Information Table S1. The initial configurations were prepared using the PACKMOL software package.^78^

The AOT and CTAB surfactant molecules were described by the general Amber force field (GAFF)^79^ with AM1-BCC^80^ charges obtained from ANTECHAMBER,^79^ and the water-filled pools were initially simulated with the TIP3P water model.^81^ For each RM size (*W*_0_ = 9–20) and each type of surfactant, we first equilibrated each water-filled RM system for 5 ns in the NPT ensemble at 298 K and 1 atm to fix the density, and for another 5 ns in the NVT ensemble at 298 K. For the CTABr system, the Br^–^ ion was fixed during an early portion of the equilibration phase, and later was relaxed for further equilibration once the RM was stable. Then, the TIP3P water pool is replaced with either pure water or 1 M HCl solutions using the ReaxFF/CGeM water model,^62,63^ i.e., the potential surface uses electrostatic embedding to describe the interactions between the classical surfactant and the ReaxFF/CGeM aqueous pool. For each reverse micelle size, we performed 1 ns equilibration followed by 3 ns of production for two statistically independent production simulations, utilizing the last 1 ns from each simulation for statistics. Convergence was confirmed through block averaging ion density distribution and proton hopping rate measurement. LAMMPS MD software is utilized for all simulations (http://lammps.sandia.gov).^82^

## Supporting Information Available

Experimental details, including THz and dielectric spectral decomposition details; real and imaginary permittivity; computational details, including radial distribution function; composition details of the reverse micelles and raw data for simulated spectral and dynamical properties. (PDF)
