# Supplementary material for: Highly Altered State of Proton Transport in Acid Pools in Charged Reverse Micelles
Source: J Am Chem Soc. 2023 Jan 12;145(3):1826–34. doi: 10.1021/jacs.2c11331 (PMC9881006; doi:10.1021/jacs.2c11331)
Supplement: Supplementary file 1 — ja2c11331_si_001.pdf [file ja2c11331_si_001.pdf]

Supporting Information

The Highly Altered State of Proton Transport in Acid Pools in Charged Reverse Micelles

Hongxia Hao[1‡], Ellen M. Adams[2,3‡], Sarah Funke[4], Gerhard Schwaab[4], Martina Havenith[4], Teresa Head-Gordon[1,5,6*]

[1]*Kenneth S. Pitzer Center for Theoretical Chemistry, Department of Chemistry, University of California, Berkeley, California 94720, USA*
[2]*Cluster of Excellence Physics of Life, Technische Universität Dresden, 01307 Dresden, Germany*
[3]*Helmholtz-Zentrum Dresden-Rossendorf, Institute of Resource Ecology, 01328 Dresden, Germany*
[4]*Lehrstuhl für Physkalische Chemie II, Ruhr Universität Bochum, 44801 Bochum, Germany*
[5]*Department of Bioengineering, Department of Chemical and Biomolecular Engineering, University of California, Berkeley, California 94720, USA*
[6]*Chemical Sciences Division, Lawrence Berkeley National Laboratory, Berkeley, California 94720, USA*
**Corresponding author: thg@berkeley.edu*

Damped Harmonic Oscillator Fitting. THz *ΔΔα* spectra of AOT and CTAB reverse micelles were fit with five or four damped harmonic oscillator terms, respectively, as shown in Equation S1

$$\Delta\Delta\alpha(v) = \sum_{i=1}^{j} \frac{A_i \omega_i^2(v) v^2}{4\pi^3\left(\left(v_{d,i}^2 + \frac{\omega_i^2(v)}{4\pi^2} - v^2\right) + \frac{v^2\omega_i^2(v)}{\pi^2}\right)} \qquad \text{(S1)}$$

Where A_i, w_i, and v_d represent the amplitude, width, and central frequency of the ith resonance. For all fits, a damped harmonic oscillator term was fixed at 0 cm^{-1} to account for low frequency contributions (below 50 cm^{-1}).

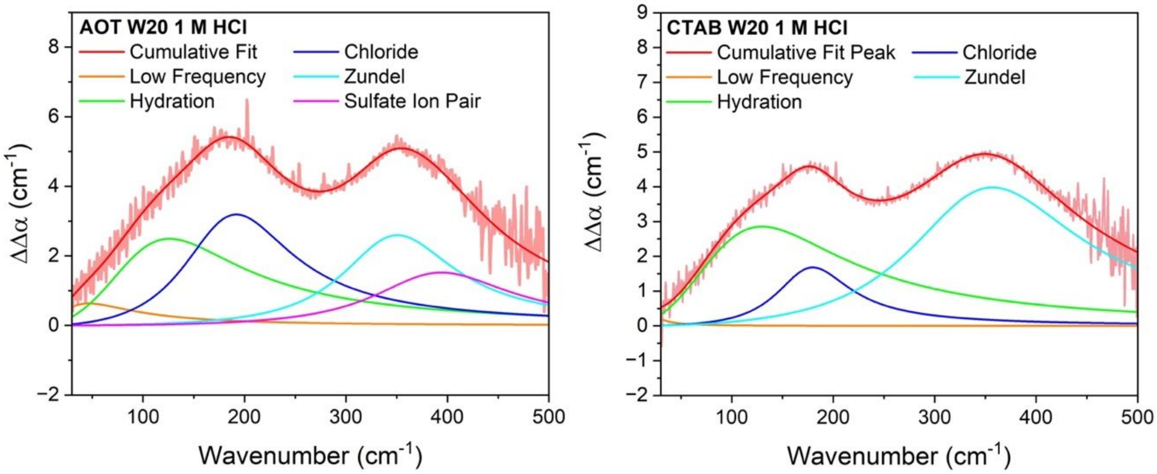

Figure S1. *Spectral decomposition of W_0=20 AOT and CTAB reverse micelles spectra with damped harmonic oscillators.* Five peaks were used to model the spectra of AOT, while four peaks were sufficient for CTAB. For all

fits, a damped harmonic oscillator term was fixed at 0 cm^{-1} to account for low frequency contributions (below 50 cm^{-1}).

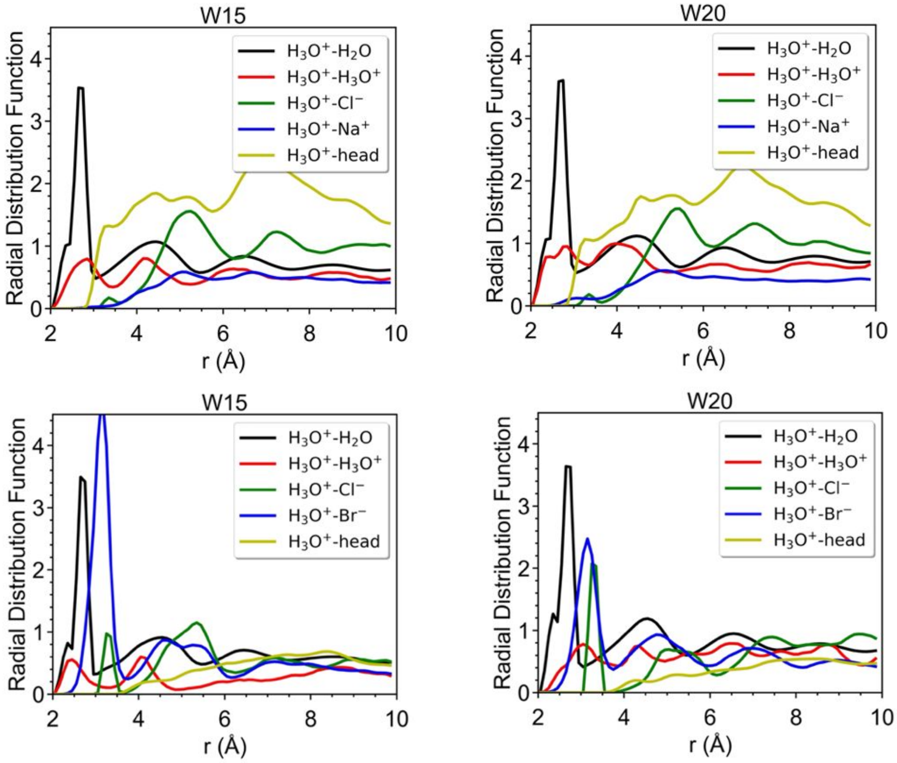

Figure S2. *Radial distribution function of hydrated protons from 1M HCl acid pools in (top) the larger NaAOT reverse micelles and (bottom) the larger CTAB reverse micelles.*

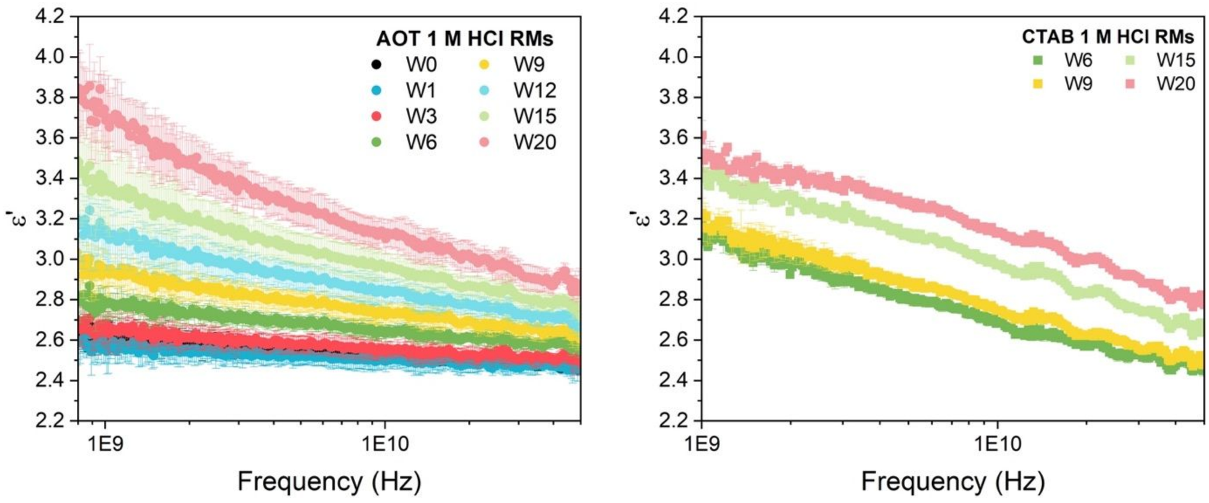

Figure S3. *Real permitivitty (ε') of AOT and CTAB RMs loaded with 1 M HCl solution.*

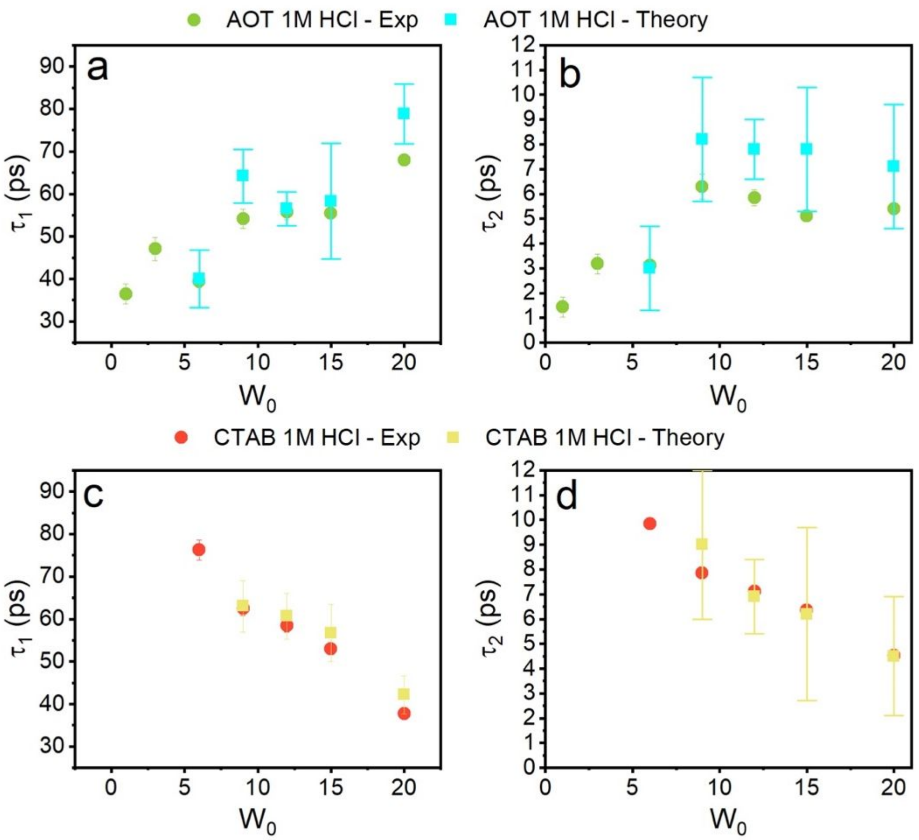

Figure S4. *Rotational relaxation times constants determined for reverse micelles containing 1 M HCl acid solution* determined from both Debye fitting of the dielectric spectra and simulation yielding (a, c) slow water (τ_1), and (b, d) fast water (τ_2) in AOT and CTAB reverse micelles respectively.

Table S1. Computational details about the compositions of the reverse micelle systems and the numerical fits to dielectric relaxation time scales of 1M HCl reverse micelles of varying sizes.

**RM**		**Water pool radius (nm)**	**Number HCl (H_20) in simulation (1 M)**	**Fitted DR τ_1 (ps)**	**Fitted DR τ_2 (ps)**
AOT	W6	1.42	7 (400)	40 ± 6.8	3 ± 1.7
	W9	1.86	16 (900)	64.2 ± 6.3	8.2 ± 2.5
	W12	2.29	30 (1678)	56.5 ± 4	7.8 ± 1.2
	W15	2.73	51 (2843)	58.3 ± 13.6	7.8 ± 2.5
	W20	3.19	82 (4536)	78.8 ± 7	7.1 ± 2.5
CTAB	W9	1.17	4 (224)	63 ± 6	9 ± 3
	W12	1.56	10 (531)	60.7 ± 5.4	6.9 ± 1.5
	W15	1.95	19 (1036)	56.7 ± 1.7	6.2 ± 3.5
	W20	2.6	44 (2456)	42.2 ± 4.5	4.5 ± 2.4

Table S2. Numerical values of the simulated forward and oscillatory proton hopping in AOT and CTAB reverse micelles for 1M HCl of varying sizes.

	**AOT**			**CTAB**		
	Forward proton hopping rate $1/\tau$ (ps^{-1})	Ratio of oscillatory to non-oscillatory hops	Residence Time (ps)	Forward proton hopping rate $1/\tau$ (ps^{-1})	Ratio of oscillatory to non-oscillatory hops	Residence Time (ps)
W6	0.033±0.003	4.6±0.4	6.5±0.02	-	-	
W9	0.027±0.001	6.3±0.6	6.3±1.0	0.004±0.005	16.2±6	8.7±0.1
W12	0.016±6E-4	5.5±0.2	11.2±0.7	0.0034±0.0015	17.4±3	14.9±2.5
W15	0.011±0.0012	6.9±0.45	13.7±0.8	0.0018±5E-4	16.1±3	32.1±2.9
W20	0.008±6E-4	7.8±0.4	15.8±0.8	0.0014±5E-4	15.1±4	60.3±1.4
